# Successful outcome of assisted reproductive technology in women with polycystic ovary syndrome combined with empty follicle syndrome: A case report

**DOI:** 10.1097/MD.0000000000043001

**Published:** 2025-06-27

**Authors:** Danyang Guo, Lin Zhang, Xiaoxia Lin, Sha Tang, Bin Zhang, Linlin Ni

**Affiliations:** a The Second Affiliated Hospital of Shandong University of Traditional Chinese Medicine, Jinan, Shandong Province, China; b Dongying People’s Hospital, Dongying, Shandong Province, China.

**Keywords:** Beinaglutide, empty follicle syndrome, obese polycystic ovary syndrome, pretreatment protocol

## Abstract

**Rationale:**

Empty follicle syndrome (EFS) is a rare and difficult-to-treat condition. Obese polycystic ovary syndrome (PCOS) is considered an independent risk factor for it. In this study, we report a case of successful pregnancy in an obese patient with PCOS combined with EFS and discuss the pathogenesis of EFS and optimal treatment strategies.

**Patient concerns:**

A 25-year-old woman presents with “6 years of infertility without contraception.”

**Diagnoses:**

Primary infertility, bilateral fallopian tube obstruction, PCOS, and EFS.

**Interventions:**

The patient underwent 2 cycles of controlled ovarian stimulation. In the first cycle, despite adequate ovarian stimulation, oocytes were not obtained and a diagnosis of EFS was made. In the second cycle, the following optimization measures were taken: the use of beinaglutide to help the patient lose weight and improve her blood glucose levels, supplementation with luteinizing hormone-active medications (e.g., Menotropins for injection), an increase in the dose of human chorionic gonadotropin, and the addition of growth hormone to improve the outcome of oocytes retrieval once the patient’s blood glucose levels had stabilized.

**Outcomes:**

In the second cycle, 15 oocytes were successfully obtained, 13 oocytes of which were Metaphase II, resulting in the culture of 5 high-quality embryos. The patient had a successful pregnancy after frozen embryo transfer and was followed up to late-term pregnancy without abnormalities.

**Lessons:**

Multiple complex mechanisms may be involved in the development of EFS, and preconditioning and individualized treatment protocols are essential for improving outcomes in patients with EFS. Our treatment protocol provides some reference basis for the treatment of EFS.

## 1. Introduction

Empty follicle syndrome (EFS) is a rare and therapeutically challenging condition,^[1]^ characterized by normal ovarian response, follicular development and serum estradiol (E2) levels as expected during controlled ovarian hyperstimulation (COH), but the absence of oocytes from the follicles at oocytes retrieval, resulting in the inability to obtain oocytes for subsequent in vitro fertilization embryo transfer (IVF-ET). EFS was originally described by Coulam et al in 1986.^[2]^ The prevalence of EFS has been estimated to range from approximately 0.045% to 7% in patients treated with IVF.^[3,4]^ Two types of EFS have been defined in the literature. True “EFS” is an inadequate response to human chorionic gonadotropin (hCG) or gonadotropin (Gn)-releasing hormone agonists and may be associated with a mutation in the inherited gene.^[5,6]^ Pseudo “EFS” is mainly due to problems related to the drug structure or methods of administration, and can be rescued by reinjecting hCG or Gn-releasing hormone agonists to obtain mature oocytes for a satisfactory pregnancy outcome. However, the mechanism of true “EFS,” which still occurs when the level of hCG is high and adequately utilized, is not clear. In this study, we report a case of a rare patient with EFS whose treatment strategy was adapted to help her achieve successful oocytes retrieval and pregnancy.

## 2. Case report

The patient was 25 years old and presented to our hospital in January 2024 due to 6 years of infertility without contraception. The patient had her first menarche at the age of 17 and had irregular menstrual cycles ranging from 30 to 90 days. The patient’s previous vaginal ultrasound showed polycystic changes in both ovaries, and there was no dominant follicular development under the natural menstrual cycle. In February 2022, the patient underwent a uterine tubal iodine-oil angiogram, which showed bilateral fallopian tube insufficiency due to obstruction. In May 2023, the patient’s serum antimüllerian hormone level was 2.8 ng/mL, which was suggestive of a good ovarian reserve function. In June 2023, the patient’s laboratory examination revealed the following serum hormonal concentrations: basal Luteinizing hormone (LH) 11.34 IU/L and basal follicle stimulation hormone (FSH) 8.84 IU/L. The patient’s height was 165 cm, body mass was 127.6 kg and body mass index (BMI) was 46.9 kg/m^2^. Ancillary tests showed that in January 2024, the patient’s abdominal ultrasound showed mild fatty liver; fasting blood glucose (FBG) level was 6.17 mmol/L; fasting insulin (FINS) level was 23.76 mIU/ml; glycated hemoglobin level was 6.2%; and there were no significant abnormalities in the liver and kidney function levels. Her husband’s semen was normal. The peripheral blood cells of both spouses had normal karyotypes. The patient was recommended to undergo IVF-ET because of “primary infertility, bilateral fallopian tube obstruction and polycystic ovary syndrome.”

### 2.1. The first-cycle IVF antagonist protocol

On the 2nd day of menstruation, the patient’s basal sex hormones showed: FSH, 8.27 mIU/mL; LH, 9.92 IU/L; E2, 252.14 pmol/L; and progesterone (P), 1.84 nmol/L. Vaginal ultrasound showed about 12 sinus follicles with a diameter of 3 mm in each of the bilateral ovaries, and uFSH (75 IU, Livzon Pharmaceutical Factory, China) then was given to initiate the process with 300 IU per day. On the 6th day of menstruation, her sex hormones showed: LH, 5.12 mIU/L; E2, 318.99 pmol/L; P, 2.09 nmol/L; and recombinant human lutropin alfa for injection (Merck, Germany) 75 IU per day was added. On the 10th day of menstruation, vaginal ultrasound suggested that the patient’s dominant follicle was up to 13 mm in diameter bilaterally, and the sex hormone levels showed: LH, 7.44 mIU/L; E2 1596.84 pmol/L; P, 2.64 nmol/L; thus Gn-releasing hormone antagonists (MerckSerono, Switzerland) 0.25 mg per day were added. On the 15th day of menstruation, sex hormones showed: LH, 14.21 mIU/L; E2, 6677.04 pmol/L; P, 6.26 nmol/L; and vaginal ultrasound showed a total of 9 follicles with a diameter of ≥ 14 mm bilaterally. Therefore Gn-releasing hormone antagonists was changed to 0.5 mg, and 6000 IU of hCG (Livzon Pharmaceutical Factory) was used to retrieve oocytes by transvaginal puncture after 36 hours. Six dominant follicles were found in the left ovary by puncture, but no oocytes were found in the follicle flushing for 5 times, which was considered to be the result of the operation error during the process of oocytes retrieval and the operation had to be stopped. The patient’s serum β-hCG was showed 110.35 mIU/mL. Two hours later, a physician with more experience in oocyte recovery performed the operation and punctured 5 dominant follicles on the right ovary, but after repeated rinsing, no oocytes still were seen. Therefore, this cycle was canceled.

Given that the patient did not obtain oocytes in the previous IVF cycle, there was still a risk of empty follicle in further IVF cycle. After fully communicating with the patient, the patient’s body mass and blood glucose level were first pretreated, and the relevant examinations were further improved and the ovulation promotion protocol was adjusted with a view to improving the patient’s outcome of assisted conception.

In February 2024, the patient returned to the clinic, and her body mass was 130 kg, after full communication with the patient, the patient’s lifestyle was instructed, and at the same time, the pretreatment of 0.12 mg/d Beinaglutide (Benemae Pharmaceutical Factory, China) was given to assist in weight loss. After 1 month of treatment, the patient’s FBG and FINS levels were normal, and her body mass was 122 kg. During the medication period, the patient did not see any obvious drug side effects.

### 2.2. The second-cycle IVF antagonist protocol

The patient underwent the second cycle of IVF antagonist protocol in March 2024, with Gn-releasing hormone agonists (Triptorelin Acetate, GenSci Pharmaceutical Factory, China) 0.1 mg and human menopausal gonadotropin (Livzon Pharmaceutical Factory) 300 IU per day initiated on the 3nd day of menstruation, and recombinant human growth hormone for injection (Anhui anke biotechnology Factory, China) 6 IU per day added until the trigger day. A total of 13 days of Gn was used, with a total of 3900 IU of Gn. On the 15th day of menstruation, the patient’s serum hormone results showed: LH, 8.44 mIU/L; E2, 8286.34 pmol/L; P, 1.97 nmol/L; and the ultrasound results showed: endometrium thickness of 16 mm with strong echogenicity, and a total of 15 dominant follicles in the ovaries bilaterally. Therefore, the patient was injected with recombinant-hCG (Merck) 250 µg combined with hCG (Livzon Pharmaceutical Factory) 2000 IU, next the oocytes were retrieved by vaginal puncture 38 hours later. A total of 15 oocytes were obtained, including 13 oocytes of Metaphase II stage, and 8 two pronuclus were fertilized. A total of 5 high-quality embryos were cultured, including 2 cleavage stage embryos (all with embryo grading of 8C I) and 3 blastocysts (all with embryo grading of 4AA). The patient’s fresh embryo transfer was canceled due to endometrial factors and all embryos were frozen.

### 2.3. Frozen embryo transfer cycle

In May 2024, the patient’s body mass was 121 kg, and continued to receive daily injections of 0.12 mg of Beinaglutide for 2 months to assist with weight loss. In June 2024, the patient underwent hysteroscopic endometrial electrosurgery with postoperative antibiotic therapy for 2 weeks.

In July 2024, the patient weighed 110 kg, with a total weight loss of 20 kg, an artificial cycle protocol was used to prepare the patient’s endometrium, and estradiol valerate tablets (Bayer Company, France) were given at 6 mg per day. A vaginal ultrasound was performed on 23 July, showing that endometrium was 9 mm. Progesterone injections were given to transform the endometrium. Two cleavage stage embryos were transferred on July 26, 2024, the patient’s serum β-hCG showed 180 IU/L on August 7, 2024, the patient underwent ultrasound which showed early intrauterine pregnancy (twin pregnancy) on August 16, 2024. On February 10, 2025, the patient’s blood glucose and blood pressure levels were under stable control, and the ultrasound suggested late twin pregnancy (consistent with 30 + weeks, double chorionic and double amniotic).

## 3. Discussion

This study reports a case of successful pregnancy in a patient with EFS. The patient underwent 2 cycles of COH. During the first cycle, despite adequate ovarian stimulation, no oocytes were retrieved and the diagnosis of empty follicle syndrome was made. The occurrence of this condition can be very challenging for both the clinician and the patient.^[7]^

### 3.1. The pathogenesis of EFS is complex and multifactorial

The development of EFS may involve a number of complex mechanisms that interact with each other to result in the failure of oocytes to mature and shed properly. Firstly, despite normal hormonal response and satisfactory hCG bioavailability, oocytes may have formed early atresia in the follicle, leading to abnormal oocyte biology in the removed oocyte.^[8]^ Secondly, genetic mutations may play an important role in the pathogenesis of EFS. Mutations in the Luteinizing hormone/chorionic gonadotropin receptor (LHCGR) gene cause partial or complete loss of response to LH, resulting in LH resistance.^[9–11]^ This mutation affects LHCGR glycosylation, protein levels, subcellular localization and triphosaden depletion, which in turn affects its signal transduction. Lu et al reported a successful treatment of patients with LHCGR mutations. Using a combination of transvaginal ultrasound and adjusted HMG doses, they removed oocytes and obtained high-quality embryos from 3 women with LHCGR mutations. Two of them had successful pregnancies and live births.^[12]^ In addition, polycystic ovary syndrome (PCOS) has been shown to be an independent risk factor for EFS,^[13]^ the mechanism of which may be related to persistently elevated LH levels in the basal state as well as deficient or delayed expression of the LH receptor. Patients with PCOS combined with obesity have a significant increase in fatty acid metabolites in the follicular fluid, a high-fat microenvironment which is not conducive to follicular development and affects oocyte maturation and fertilization. This high-fat microenvironment is detrimental to follicular development, affecting oocyte maturation and fertilization.^[14]^ At the same time, abnormally high leptin levels in follicular fluid and insulin resistance may interfere with the normal differentiation of ovarian granulosa cells and even inhibit follicular growth and development, leading to premature follicular atresia and apoptosis.^[15]^

### 3.2. Optimization of treatment strategies is essential to improve the outcome of EFS

In this case, the reasons for the failure of the first cycle may be attributed to the following aspects. Firstly, the LH threshold of follicular in PCOS patients was high compared to normal women of reproductive age, and this abnormality may have impeded the early follicular development process, which in turn affected the normal maturation of oocytes.^[16,17]^ Secondly, obesity and glucose metabolism disorders affect the drug concentration of hCG, which fails to reach a level sufficient to stimulate the ovarian response, resulting in an inadequate ovarian response to hCG.^[17]^ Gambini et al showed that higher BMI levels affect the uptake of triggering drugs, and there is a correlation with the state of insulin resistance in the ovary, which may promote premature atresia and apoptosis, interfering with the maturation of oocytes.^[18]^ Finally, the insufficient dose of the trigger drug is also an important influence. These reasons make the oocytes not fully exposed to the action of hCG and unable to detach from the granulosa cells and follicular wall to mature and ovulate.

To address these issues, we took the following steps in the second cycle. In infertile patients with PCOS combined with obesity, several guideline consensus have emphasized that weight management prior to undergoing IVF-ET is essential for achieving good clinical pregnancy and maternal and fetal outcomes.^[19,20]^ Moreover, the recommendations from the 2023 International Evidence-based Guideline for the Assessment and Management of PCOS has clarified for the first time that for PCOS patients with BMI ≥ 25 kg/m^2^, weight management can be performed in combination with glucagon-likepeptide-1 analogues if lifestyle interventions are ineffective.^[21]^ Therefore, we first used Beinaglutide was to help patients lose weight and improve FBG levels, thus optimizing the ovarian microenvironment. Clinical studies have shown that by stimulating the expression of glucagon-likepeptide-1 receptor and insulin signaling pathway, Beinaglutide can not only effectively reduce the weight and waist circumference of overweight and obese and type 2 diabetes mellitus patients, but also improve their blood glucose level.^[22]^ Animal experiments have further demonstrated that Beinaglutide can reduce weight gain and fat storage induced by a high-fat diet, as well as attenuate the abnormalities of lipid and hormone profiles associated with obesity.^[23]^ Additionally, it has been documented that Beinaglutide also reduces total and free testosterone levels in obese patients with PCOS, contributing to the alleviation of hyperandrogenic state.^[24]^ Meanwhile, the half-life of Beinaglutide is only 11 minutes, and it can be basically excreted from the body in about 90 minutes after stopping the drug, with rapid elimination and no accumulation, which further reduces the potential risk of long-term use for patients. The feature of Beinaglutide also provides pregnant patients with the possibility of direct pregnancy without intervals after withdrawal of the drug, shortening the time to pregnancy as much as possible. Secondly, increasing the trigger dose of hCG and prolonging the time between trigger and oocytes retrieval ensured that oocytes were adequately exposed to the effects of hCG. Scholars such as Abdalla^[25]^ have reported that β-hCG levels increased as the trigger drug dose was increased, which improved the rate of oocytes retrieval. Our results indirectly confirmed the evidence of scholars such as Abdalla. In addition, Abdalla’s study found a dose-dependent relationship between the shedding of the ovarian mound-oocyte complex with granulosa cells and follicular walls during ovarian stimulation cycles and hCG, with patients who failed to retrieve oocytes with 2000 IU hCG successfully obtaining oocytes in subsequent cycles with 5000 IU or 10,000 IU hCG. In patients with EFS, it may take longer for oocytes to detach from the follicular wall for oocyte mound expansion, and the rate of egg retrieval increases as the trigger time increases with oocytes retrieval. In addition, supplementation with LH-active drugs (e.g., HMG) to supplement potential LH deficiency promotes oocyte maturation.^[26]^ Finally, growth hormone was added after the patient’s blood glucose level was stabilized to increase ovarian response, promote follicular development and improve oocyte quality.^[27]^ These combined measures ultimately succeeded in improving the rate of oocyte acquisition and achieving pregnancy.

### 3.3. Importance of pretreatment and individualized treatment

Jin et al reported a 0.12% incidence of EFS in PCOS patients.^[28]^ In contrast, the occurrence of EFS in the previous cycle predicted a 20% risk of recurrence of another EFS.^[29]^ Therefore, patients with a history of previous EFS should be informed of the risk of recurrence of the syndrome and of reduced pregnancy rates in subsequent IVF cycles. This case highlights the importance of pretreatment prior to controlled ovarian stimulation. Helping patients to control their blood glucose, insulin, and body mass is key to improving the success of treatment. In addition, the development of an individualized treatment plan should be based on the patient’s specific circumstances, including PCOS status, degree of obesity, and glucose metabolism. In patients with a previous history of EFS, special attention needs to be paid to pretreatment before COH, adjustment of ovulation induction drugs, trigger methods and dosage to improve oocytes retrieval outcomes. Future studies need to further explore the pathogenesis of EFS in order to develop more effective treatment options, especially in genetic screening and individualized drug dose adjustment.^[29,30]^

The shortcomings of our study are that firstly the patient had only once history of EFS and was not genetically screened. Secondly, although the patient’s FBG and FINS levels were adjusted to the normal range and her body mass was reduced by 15% before entering the IVF cycle, her BMI was still at a high level. In this case, single blastocyst transfer was considered to effectively reduce the rate of multiple pregnancies and the associated risk of pregnancy complications, and to avoid the potential harm to maternal and infant health caused by twin pregnancies as much as possible. In the future, for patients with obese PCOS with metabolic abnormalities who have a history of EFS, more attention should be paid to the assessment of the quality of embryos to reduce the incidence of multiple pregnancies, and the patients should be closely followed up until live birth to ensure the health of the mother and child. In addition, fewer studies have been conducted on embryo transfer with in vitro fertilization after the pretreatment of Beinaglutide, and this study is not sufficient to form a definitive conclusion, but only provides a clinical reference for the diagnosis and treatment of this type of patients, as well as for the impact on the early development of the offspring and metabolic indexes in the long term, which still needs to continue to be monitored.

## 4. Conclusion

In this study, we successfully treated an obese patient with PCOS combined with EFS, using pretreatment and personalized controlled ovulation protocol to help the patient to reduce her weight, improve her metabolism, increase the number of oocytes retrieval, and enter into the endometrium preparation cycle successfully, shortening the time to pregnancy. This study provides a reference for the diagnosis and treatment of assisted reproductive technology in this type of patients, but a more standardized diagnosis and treatment process and a large-scale case study are still needed to further explore this issue in the future.

## Author contributions

**Conceptualization:** Danyang Guo, Lin Zhang, Xiaoxia Lin.

**Data curation:** Danyang Guo, Xiaoxia Lin.

**Formal analysis:** Lin Zhang, Sha Tang.

**Funding acquisition:** Linlin Ni.

**Investigation:** Lin Zhang.

**Methodology:** Sha Tang.

**Project administration:** Linlin Ni.

**Supervision:** Bin Zhang.

**Validation:** Bin Zhang.

**Visualization:** Xiaoxia Lin, Sha Tang.

**Writing – original draft:** Danyang Guo.

**Writing – review & editing:** Danyang Guo, Lin Zhang.
